# Identification of genes involved in interactions between *Biomphalaria glabrata* and *Schistosoma mansoni* by suppression subtractive hybridization

**DOI:** 10.1016/j.molbiopara.2006.09.009

**Published:** 2007-01

**Authors:** Anne E. Lockyer, Jennifer Spinks, Leslie R. Noble, David Rollinson, Catherine S. Jones

**Affiliations:** a Wolfson Wellcome Biomedical Laboratory, Department of Zoology, The Natural History Museum, Cromwell Road, London SW7 5BD, UK; b School of Biological Sciences, Aberdeen University, Tillydrone Avenue, Aberdeen AB24 2TZ, UK

**Keywords:** *Biomphalaria glabrata*, *Schistosoma mansoni*, Suppression subtractive hybridization, SSH, Ferritin, HtrA2, Resistance

## Abstract

*Biomphalaria glabrata* is an intermediate snail host for *Schistosoma mansoni*, a medically important schistosome. In order to identify transcripts involved in snail-schistosome interactions, subtractive cDNA libraries were prepared, using suppression subtractive hybridization (SSH) between a parasite-exposed schistosome-resistant and a susceptible strain of *B. glabrata*, and also between schistosome-exposed and unexposed snails from the resistant snail line. Separate libraries were made from both haemocytes and the haemopoietic organ. Subtraction was performed in both directions enriching for cDNAs differentially expressed between parasite-exposed resistant and susceptible samples and up or down-regulated in the resistant line after challenge. The resulting eight libraries were screened and eight genes, differentially expressed between the haemocytes of resistant and susceptible snail strains, were identified and confirmed with reverse transcriptase PCR, including two transcripts expected to be involved in the stress response mechanism for regulating the damaging oxidative burst pathways involved in cytotoxic killing of the parasite: the iron-storage and immunoregulatory molecule, ferritin, and HtrA2, a serine protease involved in the cellular stress response. Transcripts with elevated levels in the resistant strain, had the same expression patterns in the subtracted libraries and unsubtracted controls; higher levels in exposed resistant snails compared to susceptible ones and down-regulated in exposed compared with unexposed resistant snails. Differential expression of two of the transcripts with no known function from the susceptible strain, was independently confirmed in a repeat exposure experiment.

## 1 Introduction

The freshwater snail *Biomphalaria glabrata* is an intermediate host for the digenean trematode *Schistosoma mansoni*, which causes human intestinal schistosomiasis. It is also an important model invertebrate and currently the subject of a large-scale genome project (see http://biology.unm.edu/biomphalaria-genome/). Interactions between snails and schistosomes are complex and there is a need to elucidate pathways involved in snail-parasite interactions and identify those factors involved in the intricate balance between the snail internal defence system (IDS) and trematode infectivity mechanisms that determine the success or failure of an infection (for a review see [1]). Compatible snail-trematode infections often reflect the parasite's capacity to avoid or interfere with the innate response of the snail. The term ‘resistant’ can be applied to those individuals within a single snail species that are able to evade infection by a species or strain of schistosome that is normally capable of parasitizing that species of snail.

Relatively little is known of the molluscan IDS compared to vertebrate immune systems. Molluscs appear to lack an adaptive immune system like that found in vertebrates and, instead, are considered to use various innate mechanisms involving cell-mediated and humoral reactions that interact to recognize and eliminate invading pathogens or parasites in incompatible or resistant snails (for reviews see [2], [3], [4]). However, a diverse family of fibrinogen related proteins (FREPs) containing immunoglobulin-like domains has been discovered in *B. glabrata* and may play a role in snail defence [5]. Circulating haemocytes (macrophage-like defence cells) in the snail haemolymph are known to aggregate in response to trauma, phagocytose small particles (bacteria and fungi) and encapsulate larger ones, such as trematodes. Final killing is effected by haemocyte-mediated cytotoxicity mechanisms involving non-oxidative and oxidative pathways, including lysosomal enzymes and reactive oxygen/nitrogen intermediates [6], [7].

Despite many expression analyses aimed at identifying defence related genes involved in snail-trematode interactions, including subtractive hybridization [8], differential display [9], [10], [11], [12], [13], [14] and expressed sequence tag programmes [15], [16], much remains to be discovered concerning specific molecules mediating the defensive events in snail intermediate hosts, in particular the response shown by resistant or incompatible snails. In this study a sensitive, widely used PCR-based subtraction approach, suppression subtractive hybridization [17] and a PCR technique for amplifying the initial cDNA samples [18], were used to investigate how *B. glabrata* responds to *S. mansoni* infection. Previous SSH studies in *B. glabrata* examined the whole head-foot region [19], however, with the combined PCR approach it was possible to examine individual tissues within the snail and focus on haemocytes, the primary effector cells involved in the snail immune defence and the haemopoietic organ, responsible for producing the haemocytes. SSH allows a comparison of two mRNA pools simultaneously and the experiments were chosen to investigate gene expression in resistant snails. Parasite-exposed resistant and susceptible snail lines were compared as well as exposed and unexposed resistant snails in order to see which genes are up or down-regulated in response to exposure to the parasite and to identify genes involved specifically in the defence response of resistant snails.

## 2 Materials and methods

### 2.1 Snail material and parasite exposure

The first experiment used 60 adult *B. glabrata* snails from susceptible strain (NHM Accession number 1742) and 60 from resistant strain (NHM Accession number 3017, derived from BS-90 [20]). The snails were held overnight in autoclaved snail water with 100 μg/ml ampicillin to minimize bacterial contamination. Each snail was individually exposed to 10 schistosome miracidia (Belo Horizonte strain). This experiment was also repeated to test the expression of identified transcripts. A second experiment used 120 snails from the resistant snail strain, but only 60 of the snails were exposed to the parasite as described before, while 60 were kept unexposed in identical conditions as a control. The sampling regimes for both experiments were the same. Twelve snails from each sample group were taken at 5 time periods, 2, 4, 6, 8 and 24 h after exposure to the parasite. The material from each time point was pooled to give sufficient RNA for the SSH and the extended sampling regime was necessary to allow time for the snails to be dissected. This also meant that all the transcripts that are expressed over the first 24 h after infection would be obtained. Snails were swiftly killed by decapitation, and all the exuded haemolymph collected. The haemolymph was pooled for each sampling time and snail strain and the haemocytes pelleted by spinning at 10,000 × *g* at 4 °C for 20 min. The pellet was frozen in liquid nitrogen and stored at −80 °C. Snails were preserved in 1 ml RNAlater (Ambion Inc., Texas, USA) and stored at 4 °C until dissection. The haemopoietic organ, responsible for producing haemocytes that have an active role in the snail internal defence system (reviewed in [6]) was dissected for each snail (preservation in RNAlater makes this organ easy to identify and dissect cleanly from surrounding tissues) and pooled for each strain from all time points.

### 2.2 RNA extraction and SMART cDNA synthesis

Total RNA was extracted from haemopoietic organ or haemocyte pools using the SV RNA extraction kit (Promega UK Ltd., Southampton, UK) according to the manufacturer's protocol. This kit includes DNAse treatment to eliminate genomic DNA contamination. cDNA synthesis and subsequent amplification was performed using 1 μg total RNA with the SMART™ PCR cDNA synthesis kit (CLONTECH) according to the manufacturer's instructions.

### 2.3 Suppression subtractive hybridization and library construction

Eight independently subtracted libraries were made using the PCR select kit (BD Clontech). These were resistant minus susceptible and vice versa for both the haemopoietic organ and haemocytes from the first experiment and exposed minus unexposed snails of the resistant line and vice versa from the second experiment again both from the haemocytes and haemopoietic organ. The subtraction was carried out as described in the kit manufacturer's instructions. However, six primary amplification PCRs, followed by a secondary PCR from each primary PCR were carried out and then these PCRs were pooled for library construction. This multiple sampling of each subtracted pool was designed to minimize sampling effects, whereby transcripts may be present or absent by chance. The libraries were made by ligating 3 μl of each pool of secondary PCRs into pGem-T easy vector (Promega) and transforming JM109 cells according to the manufacturer's instructions. The entire library was plated out onto 10 LB agar plates. From each library 2× 96 white colonies were selected for overnight culture.

### 2.4 Differential screening

Duplicate filters were made for each library by spotting 2× 96 clones grown overnight in LB, onto Biodyne B membrane (PALL) using dot-blotting apparatus (ATTO Corp. Tokyo, Japan). The membrane was soaked in 2× SSC prior to loading in the press. Then 50 μl 2× SSC with dilute bromophenol blue was sucked through followed by 20 μl LB culture. The bacteria were then denatured with 50 μl denaturing solution (1.5 M NaCl, 0.5 M NaOH), then neutralized with 50 μl of 1.5 M NaCl, 0.5 M Tris pH 7.5 solution for 2 min, and rinsed in 50 μl 2× SSC. The filters were air dried and stored at RT.

For each pair of duplicate filters, one was probed with the library from which it was made, and one with the reciprocal subtracted library, so for example the haemocyte resistant specific library filters were probed with the haemocyte resistant specific library as a probe, as well as using the susceptible haemocyte library as a probe. In each case the probe used was 10–15 μl of cleaned secondary PCR, previously digested with the restriction enzymes, *Sma*I and *Rsa*I (Boerhinger Mannheim) to remove adapter sequences and labelled with digoxigenin using DIG High Prime DNA labeling detection starter kit II (Roche). The filters were visualized by autoradiography. Positive spots were identified as those that were clearly present on the filter probed with the corresponding library while completely absent from the reverse subtracted library.

### 2.5 Sequence analysis

All identified fragments were sequenced using BigDye and run on an ABI 377. The sequence traces were examined using Sequencher and all vector and adaptor sequences removed. Sequences were compared to GenBank using BlastN BlastX and tBlastX using the program Seqtools (http://www.seqtools.dk/.) All sequences were submitted to GenBank (Accession numbers DY523246–DY523271).

### 2.6 Semi-quantitative RT-PCR

PCR was carried out using the amplified fractions as a template; this confirms the presence or absence of the fragment in the enriched libraries. PCR was also carried out on the un-subtracted control fractions—these consist of material that was amplified from the same experiment, but which was not subtracted. It was necessary to use amplified material for these PCRs due to RNA being limited.

For each identified candidate specific primers were designed and synthesized. Semi-quantitative PCR used 0.4 μM Forward and Reverse primers (see supplementary material), 1× advantage polymerase buffer, 0.2 mM dNTPs and 0.4 μl 50× advantage polymerase (BD Clontech). Cycling conditions were 94 °C for 30 s then cycles of 94 °C for 30 s, 58 °C for 30 s and 72 °C for 30 s. PCRs were cycled for a total of 33 cycles, but 5 μl of each reaction was removed after 18, 23, and 28 cycles to provide a semi-quantitative result—at lower cycle number the product in the reaction is proportional to the amount of starting material allowing us to ascertain if the levels differ, more effectively than after full cycling. These were run together on a 2% agarose gel to allow a semi-quantitative estimate of the presence of the transcript in the template. Semi-quantitative RT-PCR of actin (using *B. glabrata* specific actin primers F: TGGTGCCTTTTCTTCTCTAATTGC; R: GAAAGTGTGATGCCAGATCTTCTC) was used to assess the comparability of samples for the unsubtracted control material to confirm that equivalent quantities of cDNA had been used for the specific primers. PCR and cycling conditions were the same as for specific PCRs. Since the subtracted libraries had non-differentially expressed genes (such as actin) subtracted from them, it is not possible to use this type of control for these samples.

For the repeat experiment, 1 μl cDNA (prior to SMART amplification) was used and RT-PCR performed as above. For four of the transcripts, no amplification products were obtained from this cDNA so SMART-amplified cDNA was used as above. Actin PCRs were carried out simultaneously on both unamplified and SMART-amplified cDNA as a control. To assess statistically differential expression of these transcripts, integrated optical density (IOD) measurements were obtained for each amplified band, as a proxy for gene expression (Labworks™, Ultra Violet Products, Cambridge), and a matched paired t-test performed for each transcript, using log IOD values normalized to actin.

## 3 Results

### 3.1 Differential screening of cDNA libraries

SSH was used to make eight subtracted libraries; forward and reverse subtraction of resistant and susceptible material from haemocytes and the haemopoietic organ made two resistant-specific and two susceptible-specific libraries, one for each tissue type; and from the second experiment, forward and reverse subtraction of parasite-exposed and unexposed material from the resistant line, again from the same tissue types, made two exposed-specific and two unexposed-specific libraries. Differential screening of the 8 subtracted libraries identified 24 differentially expressed positive candidate transcripts, all of which were sequenced. These candidates were selected by presence or absence on the filters (see supplementary data), quantitative differences were ignored since the filter contained spotted bacterial colonies rather than quantified DNA. Sequencing revealed 5 clusters (2 containing 6 clones each and 3 with 2 clones each), and 6 unique sequences, resulting in 11 different candidate clusters or sequences.

Nine of the candidate sequences originated from haemocyte material, eight of which were derived from the first experiment comparing exposed resistant and susceptible snail strains. Five different sequences from the resistant minus the susceptible library were identified; Clusters 2–5 and one single sequence ZB9413. Three positives were identified from the susceptible minus resistant library, two unique sequences (ZB9365 and ZBA105) and one cluster of 6 sequences (Cluster 1), which are transcripts specific to the haemocyte susceptible library. A single transcript (ZBA3283) was identified from the second experiment of resistant-exposed minus resistant-unexposed library.

Only 2 clear positive transcripts were obtained from the haemopoietic organ material, one (ZBA2946) from the resistant unexposed minus exposed library, and one (ZB9039) from the resistant exposed minus susceptible exposed library.

### 3.2 Blast results

All the sequences were compared to the non-redundant and expressed sequence tag (EST) sections of GenBank using BlastN, BlastX and tBlastX (for details see supplementary material). Using the criteria of an *E* value of <1 × 10^−4^ for a significant match, only four had significant BLAST hits in the non-redundant section of GenBank. Cluster 5 (six sequences) identified soma ferritin from *Lymnaea stagnalis* [21], both at the nucleotide and protein level, as well as a number of other ferritin heavy chain sequences. Cluster 4 (two sequences) identified a fibrinogen related protein 3.2 from *B. glabrata* (AY028461) [22], but only at the nucleotide level—BlastX searches produced no matches. The section of sequence identified is within intron 3 (bases 6282–6112) of this protein as it is described on GenBank. However a BlastN search of ESTs identified nine other *B. glabrata* ESTs, and since this transcript also contained a poly(A) tail (more than 26 bp long), a poly adenylation signal (24 bp upstream from the start of the polyA sequence) and a 147 amino acid long open reading frame, the sequence is definitely derived from mRNA. The other three sequences from the resistant specific library identified no significant matches in the non-redundant section of GenBank, but all identified previously sequenced *B. glabrata* transcripts from dbEST. Cluster 2 initially identified 34 *B. glabrata* ESTs, but the resulting contig identified a further 11 ESTs producing a final 1493 bp contig composed of 45 ESTs, all Open Reading frame ESTs (ORESTES) [23] from both susceptible and resistant snail lines, from whole body and ovotestis. This extended sequence still produced no other significant database matches, but contained a 369 aa long open reading frame (from 1 to 1107 bp). Cluster 3 identified 46 ESTs, all from haemocyte libraries [16] apart from one fragment (CB350578) identified from a differential display experiment comparing the effect of *S. mansoni* and *Echinostoma caproni* excretory-secretory products on Bge cells, but not investigated further in that study [14]. The resulting 619 bp contig did not identify any further *B. glabrata* ESTs and the slightly extended sequence had no additional significant hits. Of the three sequences from the susceptible library, only one (ZBA105) identified a sequence on the database, but this was a putative hypothetical protein from *C. elegans*, which provides no information as to the function of this protein. The sequence from the exposed-specific library from the resistant snail line, ZBA3283, did not produce a significant hit, but examining both the BlastN and BlastX results showed that all the sequences identified were mitogen activated protein kinase (MAPK), based on the first 120 bases of the transcript fragment. This translated section identified the 3′ section of the catalytic domain of serine/threonine kinase with a BlastP search at NCBI entrez Blast interface (http://www.ncbi.nlm.nih.gov/entrez) (*E* value = 0.001) and suggests that the transcript fragment obtained from the SSH (which has a polyA tail) contains only the very end of the coding region of MAPK followed by the 3′ UTR, and this coding section was insufficient to produce a significant Blast result. It seems probable that this is a MAPK-like sequence but more sequence would be required to confirm its homology to MAPK. One of the transcript fragments (ZB9039) from the haemopoietic organ showed no significant database match, while the other (ZBA2946) was significantly similar to a serine protease HTRA2, from many organisms including mouse and human [24].

### 3.3 Confirmation of differential expression

**Fig. 1:**
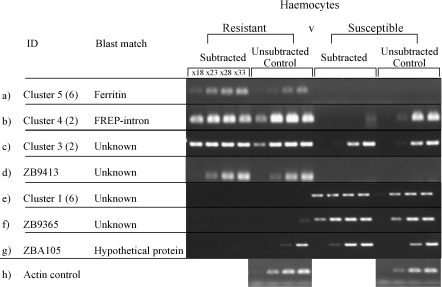
Semi-quantitative RT-PCRs to confirm differential expression of seven transcripts from haemocyte libraries. RT-PCRs were carried out on subtracted material amplified to make the libraries and unsubtracted controls, representing the original pool of transcripts, before subtraction.

**Fig. 2:**
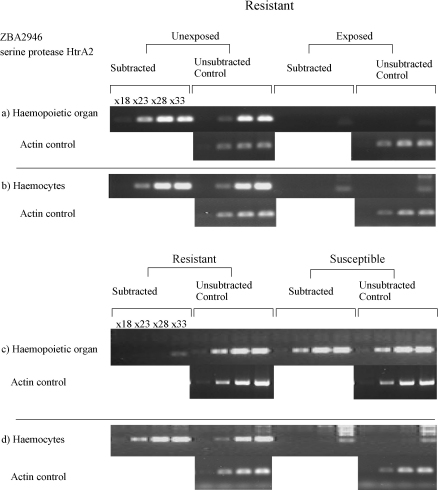
Semi-quantitative RT-PCRs of one candidate ZBA2946, homologous to HtrA2 (serine protease), in (a) haemopoietic organ unexposed-specific library from which it was identified, (b) haemocyte unexposed and exposed libraries from resistant snails, (c) resistant and susceptible-specific haemopoietic organ libraries and (d) resistant and susceptible-specific haemocyte libraries.

Semi-quantitative RT-PCR of the enriched pool confirmed differential expression for 8 of the 11 fragments (Fig. 1, Fig. 2a), justifying the stringent selection criteria used to select candidates from the filters. Specific PCRs for each identified transcript were carried out to determine the expression of each not only in the subtracted fraction, which consists of the enriched pool of products, but also in an unsubtracted control, which represents the original unsubtracted pool of transcripts. This amplified control fraction was used as only very small amounts of RNA could be obtained from the tissues used in these experiments.

Seven of the nine transcripts obtained from haemocyte material were confirmed as differentially expressed, including four derived from resistant-specific library and all three from the susceptible-specific library (Fig. 1). Cluster 2 from the resistant minus susceptible library and Clone ZBA3283 (putative MAPK-like sequence) obtained from the second experiment of resistant exposed minus resistant unexposed library could not be confirmed as differentially expressed (results not shown). For both ferritin and ZB9413 the products are noticeably present in both the subtracted fraction and the unsubtracted resistant-specific fraction and absent in both the susceptible-specific subtracted and unsubtracted fractions, demonstrating their presence in the original cDNA population from the resistant snails and absence from the susceptible one (Fig. 1a and d). Clusters 3 and 4 each show products present in the resistant subtracted fraction as well as in the original control pool, but absent or present only at higher cycle numbers in the susceptible subtracted fraction. Although there are products seen in the original unsubtracted susceptible cDNA, they were only observed at higher cycle numbers (Fig. 1b and c) indicating that although the sequences are not resistant specific, there appears to be a difference in the level of expression between the two strains. The three susceptible specific sequences, including one with a match to a putative hypothetical protein, also show confirmed differential expression (Fig. 1e–g).

Differential expression for only one out of the two transcripts obtained from the haemopoietic organ libraries could be confirmed by semi-quantitative RT-PCR. Examining the unexposed versus exposed resistant snail libraries, the serine protease fragment is clearly present in the unexposed fraction while absent from the exposed (Fig. 2a). Semi-quantitative RT-PCR of the transcript of unknown sequence, ZB9039, originating from resistant exposed minus susceptible exposed haemopoietic organ libraries did not confirm a change in expression (result not shown).

### 3.4 Characterization of differentially expressed transcripts in other subtracted libraries

Having examined and confirmed the expression patterns of eight transcripts using the libraries from which they were isolated, we also investigated the expression of these transcripts in the other independently subtracted libraries (i.e. from the other experiment and tissue type), again using semi-quantitative PCR on the subtracted and unsubtracted fractions obtained from the SSH.

#### 3.4.1 Transcripts from the resistant libraries

**Fig. 3:**
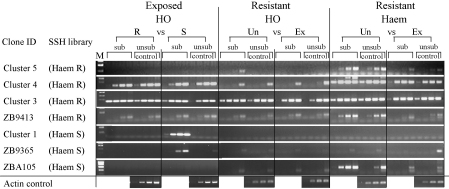
Semi-quantitative RT-PCRs showing levels of gene expression of the confirmed differentially expressed genes in the other subtracted libraries. SSH library—the library from which each fragment was originally identified. Haem: Haemocyte; HO: Haemopoietic organ; R: resistant; S: susceptible; Ex: Parasite-exposed resistant; Un: unexposed resistant. M: Marker Hyperladder IV (Bioline); sub: subtracted fraction; unsub control: unsubtracted control fraction.

The expression of the four transcripts with elevated expression in haemocytes from resistant exposed snails (compared with susceptible exposed strains) was independently examined in the exposed resistant compared with unexposed resistant subtractions (Fig. 3). This showed in all cases elevated expression in unexposed resistant snails relative to exposed snails. When expression of these transcripts from haemocyte libraries was examined in haemopoietic organ material from either resistant exposed versus susceptible exposed or exposed versus unexposed resistant libraries either no differences were detected or no PCR products were obtained for the majority of the four transcripts. Only with Cluster 3 did the material from the haemopoietic organ mirror that from the haemocytes with more product (at lower cycle number) in the material from the resistant snails compared with that from the susceptible snails. Cluster 3 also showed, again only at low cycle numbers, more product in the unexposed haemopoietic organ material compared with the exposed, in the resistant snail line.

#### 3.4.2 Transcripts from the susceptible libraries

Of the fragments with elevated transcript levels in the susceptible strain identified from haemocytes (Fig. 3) both Cluster 1 and ZB9365 showed increased expression in the susceptible enriched libraries from the haemopoietic organ, although in both cases, the transcript was not present at detectable levels in the control fractions, only after enrichment. No product was detectable in the haemocyte material from the exposed or unexposed material derived from the resistant line, but as these are susceptible specific products this is not unexpected. Only ZBA105, hypothetical protein, was detected in the resistant material and was confirmed present at higher levels in haemocyte material of unexposed resistant snails compared to exposed snails, but this fragment was not found in haemopoietic organ derived material.

#### 3.4.3 Transcripts from the unexposed libraries

The expression of ZBA2946 (serine protease HtrA2), the fragment identified from the unexposed resistant library from haemopoietic organ was also examined in the independently subtracted haemocyte material from the same experiment and this expression pattern seems to mirror that found in the haemopoietic organ material (Fig. 2b). However, on examining the resistant versus susceptible libraries, in the haemopoietic organ the susceptible and resistant controls demonstrate no difference in levels of the transcript although it is absent from the resistant specific fraction (Fig. 2c). Finally examining the material from the haemocytes, the fragment is present only in the resistant fraction, both subtracted and unsubtracted control (Fig. 2d). This means that although the serine protease HtrA2 was identified from different material, it shows the same expression patterns as ferritin and the other fragments identified from the resistant specific library, present in higher levels in the resistant specific haemocyte libraries and demonstrating elevated levels in the unexposed compared to the exposed haemocyte libraries from the resistant material.

### 3.5 Confirmation of differential expression in exposed resistant and susceptible snails

**Fig. 4:**
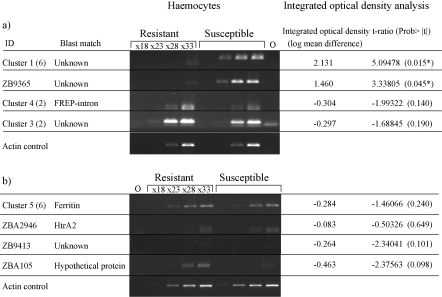
Semi-quantitative RT-PCRs with specific primers for the confirmed differentially expressed fragments on haemocytes from the repeated experiment, exposed resistant and susceptible snails. (a) PCRs using cDNA and (b) PCRs using amplified cDNA (for those fragments not detected directly from cDNA) 0—no DNA control. For each candidate integrated optical densities were calculated from the bands and compared using a matched pairs *t*-test. ^*^Significantly different expression (*p* < 0.05).

Since all of the fragments identified demonstrated differential expression between resistant and susceptible snail haemocytes, this experiment was repeated and the expression of each transcript examined in the haemocytes of these snails (Fig. 4). Using integrated optical density measurements for each band, significant differential expression was directly confirmed for two susceptible specific sequences, Cluster 1 and ZB9365, both transcripts with unknown function (Fig. 4a). Significantly different expression was not confirmed for two other sequences identified from the resistant library, Clusters 3 and 4 (FREP-intron sequence) (Fig. 4a). The other fragments could not be detected directly in cDNA from the repeated experiment (results not shown) suggesting the expression of these genes was very low. All were detected in amplified cDNA but did not demonstrate significantly different expression in this repeat experiment (Fig. 4b). Since these transcripts were all found to be down-regulated in resistant snails after exposure it seems likely that in the repeated experiment these genes were affected by parasite exposure in both the resistant and susceptible snails, making detection difficult.

## 4 Discussion

In this study, haemocyte suppressive subtractive libraries of *S. mansoni*-exposed resistant and susceptible *B. glabrata* lines were created to identify defence-relevant genes that are involved specifically in the resistant response. We also examined parasite-exposed and unexposed resistant snails to see which genes were differentially regulated in response to *S. mansoni* infection. These aims were also addressed using haemopoietic organ (tissue responsible for producing the haemocytes) SSH libraries using the same snail lines and exposures. Differential screening of 8 subtracted libraries produced 11 sequence fragments that were strong candidates for differential expression. RT-PCR using the original subtracted and control material confirmed differential expression of eight of these transcripts, seven of which were derived from haemocyte libraries. Only four had significant Blast hits in GenBank, three from haemocyte libraries (ferritin, FREP intron sequence, hypothetical protein) and one from a haemopoietic organ library (serine protease HtrA2), while three show no homology to characterized genes on GenBank, although several do identify previously sequenced ESTs with no function ascribed to them. All the fragments demonstrating elevated levels in the resistant strain, had the same expression patterns in the subtracted libraries and unsubtracted controls; higher levels in exposed resistant snails compared to susceptible ones and down-regulated in exposed compared with unexposed resistant snails. Clear differential expression of two susceptible-specific fragments (Cluster 1 and ZB9365) with no known function was shown to be repeatable.

### 4.1 Fibrinogen related proteins (FREPs)

Cluster 4 identified a section of sequence within intron 3 of one FREP molecule, a fibrinogen-related protein 3-2 precursor (GenBank accession number AY028461) [22]. FREPs are fibrinogen related proteins, which demonstrate increased expression following parasite infection, and bind to parasite antigens [22]. The sequences in Cluster 4 clearly contained a poly (A) tail, a poly adenylation signal and an 147 amino acid long open reading frame which strongly support the supposition that they are expressed genes and this suggests the interesting possibility that there may be coding regions contained in the published FREP intron sequences which should be investigated further, particularly since alternative splicing to produce truncated forms is a feature of the FREP gene family [25]. Blast searches with Cluster 4 sequence identified nine other *B. glabrata* ESTs, four of which contained this section of sequence that identified the FREP gene. No ESTs from other organisms were identified with this sequence. In some cases, however, these other ESTs did not align outside this region. Therefore another explanation for finding this sequence in the intron could be that it is a common *B. glabrata* genomic sequence motif. However, without recovering more sequence from this transcript and resolving or refuting its homology with FREP genes it is not possible to speculate further upon its function.

### 4.2 Cellular stress response proteins

#### 4.2.1 Ferritin

Cluster 5 had significant sequence homology to soma ferritin from the freshwater snail *L. stagnalis*, which has similarities to the H-subunit type of vertebrate ferritins and contains the iron-responsive element (IRE) of vertebrate ferritin mRNAs [21]. Ferritin is the major iron repository in eukaryotic cells and plays a role in regulating iron homeostasis, exhibiting a high degree of structural conservation in bacteria, plants and animals [26]. As well as iron regulation, ferritin H subunit demonstrates ferroxidase activity and may have a role as a protectant against oxygen free radical-mediated damage by limiting the amount of Fe(II) available for the generation of reactive oxygen species (ROS) [27], [28], [29], [30], [31].

Two *B. glabrata* ESTs with homology to ferritin have been previously reported. Nowak et al. [19] identified a 303 bp fragment (accession number CD760630) from a head/foot SSH library from resistant *B. glabrata* 12 h post infection with *S. mansoni* which demonstrated little differential expression, and Raghavan et al. [15] found a 492 bp EST (accession number AW739853) from a resistant strain (BS-90) haemocyte library 5 h post-exposure to *S. mansoni*. These two sequences are identical for the 303 bp, which they both contain. However, Blast analysis with Cluster 5 revealed that it was different to these ferritin-like transcripts from earlier studies; although all identified the soma ferritin from *L. stagnalis* as the closest protein match, Cluster 5 sequence had only 80% identity with CD760630 (127/158 bp) and 79% with AW739853 (127/224 bp) from the Blast results. Cluster 5 did identify two other *B. glabrata* ESTs AW740322 and AW740403 from the haemocytes of unexposed BS-90 (resistant) snails [15] with 100% and 97% identities, respectively. Ferritin H subunit genes form a multigene family [32], [33], and therefore it seems likely that we have identified a second *B. glabrata* ferritin gene. Two ferritin genes have also been identified in the oyster *Crassostrea gigas* [34], [35] and in *L. stagnalis*, [21], [36] where the sequences of both are more similar to ferritin H subunits than L type and contain H-specific amino acid residues [21].

The function of ferritin in invertebrates is less well characterized than in mammals, but it has been shown to be upregulated by LPS in echinoderms [37], in white spot syndrome virus (WSSV)-resistant shrimp compared to susceptible individuals [38], in response to anoxic conditions in *Litterina littorea* [39] and by nitric oxide (NO) in the snail *Helix pomatia* [40]. The significance of elevated levels of ferritin transcripts in resistance snails could be linked to the oxidative burst demonstrated by snails in response to parasite exposure (reviewed in [6]) since H_2_O_2_ induces ferritin in HeLa cells [31] and in *B. glabrata* the production of H_2_O_2_ demonstrated when the respiratory burst is artificially stimulated, appears greater in a resistant snail line [41]. Cytosolic copper/zinc superoxide dismutase (Cu/Zn SOD), which catalyses the conversion of superoxide to H_2_O_2_, has been shown to have a constitutive difference in mRNA levels between one mostly resistant snail line and susceptible snails [42] and a significant association of one allele of the Cu/Zn SOD gene to the resistance phenotype has been identified [43]. Therefore, given the role of ferritin in protecting against oxidative damage in vertebrates, the increased levels of ferritin observed in resistant snails when compared to susceptible, may be part of their response to parasite infection.

#### 4.2.2 Serine protease HtrA2

Clone ZBA2946 sequence showed considerable homology to a mouse serine protease HtrA2 [24]. HtrA2 (also known as Omi) removes proteins damaged or denatured after elevated temperatures or oxidative stress (reviewed in [44]). HtrA2 interacts with Mxi2 (an alternatively spliced form of p38 stress activated kinase) [45], and is thought to be involved in the cellular stress response in mammals [24].

The role of HtrA2 in *B. glabrata* is unknown, but, given the function of homologous proteins in other organisms, it may be involved in the cellular stress response. Previous findings of serine protease activity in *B. glabrata* include Bahgat et al. [46], who described trypsin-like serine protease activity from *B. glabrata* haemocytes, a finding consistent with these phagocytic cells mounting a cytotoxic attack against schistosome sporocysts. However, although the serine protease's activity varied among individual snails, there was no detectable difference between snails susceptible or resistant to schistosome infection. One EST cluster (GenBank Accession no. CK989867) identified in a haemocyte cDNA library from snails maintained in non-axenic conditions [16], exhibits a portion of the Tryp-SPc (trypsin-like serine protease), but this shows no homology to our sequence at the nucleotide level.

### 4.3 Conclusions

Both ferritin and HtrA2, as stress response genes, would be expected to be stimulated in response to schistosome challenge, and elevated levels in resistant snails are consistent with this. How then to account for the reduction in mRNA for both these genes that appears in parasite-exposed resistant snails compared to unexposed snails? One possible explanation may lie with the interaction between snail and parasite. The influence of the parasite must be considered since it manipulates the normal snail defence response, and this effect may be greater in susceptible snails than in resistant ones. It is possible that some effect of the parasite that reduces the snails’ normal response to oxidative stress occurs, but is less significant in resistant snails, allowing them to overcome the challenge. This would result in the expression patterns seen for these genes and would also account for the low levels detected in the repeated experiment.

Two identified susceptible-specific transcripts (Cluster1 and ZB9365) were independently confirmed as being present only in susceptible snails in the repeat experiment. Further characterization of these transcripts with unknown function is required to determine if these transcripts are involved in the response of susceptible snails to parasite exposure and how that response differs to that of resistant snails. Surprisingly six of the identified candidates did not show differential expression in the repeat experiment. This could be due to a number of factors, including variability in the conditions of parasite exposure, loss of signal due to pooling the samples over the 24 h time period (meaning that small or transient changes might not be detected) and the fact that for four of the samples a product could only be detected faintly after amplifying the cDNA, which again might prevent the detection of small differences in expression.

In summary, we have identified several transcripts by differential screening of SSH libraries from *B. glabrata* that are expected to be involved in the stress response mechanism for regulating the damaging oxidative burst pathways involved in cytotoxic killing of the parasite. While no single transcript is likely to be solely responsible for conferring differing susceptibility to *S. mansoni* infection, differences in gene expression demonstrate a difference in the snails’ response to infection and may indicate which mechanisms are employed by the snails in their defence response. Whilst interesting candidate genes have been found using SSH, it is not a particularly efficient method for the analysis of gene expression, especially given that many of the candidates identified in this way did not show differential expression in a repeat experiment. A different approach to transcriptome analysis, such as using microarray technology, will enable a more thorough examination of changes in gene expression in *B. glabrata*, as a much larger number of genes can be analyzed simultaneously, using smaller amounts of RNA. The SSH libraries produced in this study, as well as larger libraries from a modification of the EST strategy, termed ORESTES (Open Reading Frame ESTs; [23]), have been used to make the first version of a custom *B. glabrata* cDNA microarray. This will enable a more detailed investigation of the transcriptome in response to trematode infection in this snail intermediate host. This approach has been used successfully for expression studies of gender-associated gene transcripts in *S. mansoni* [47], [48]. Differentially expressed snail transcripts reported here are also included on the array, enabling them to undergo further screening [49] for a more thorough understanding of their role within the snail internal defence system.
