# Supplementary material for: Identification of genes involved in interactions between Biomphalaria glabrata and Schistosoma mansoni by suppression subtractive hybridization
Source: Mol Biochem Parasitol. 2007 Jan;151(1):18–27. doi: 10.1016/j.molbiopara.2006.09.009 (PMC1852639; doi:10.1016/j.molbiopara.2006.09.009)
Supplement: Supplementary file 1 [file mmc1.doc]

| Clone ID or cluster N^o^. (N^o^. sequences in cluster) | SSH-EST Accession number | Size (bp) | Poly (A) tail /  Poly (A) signal /  N^o^. bp upstream | Largest ORF (position in sequence) | Material/ Library found in | Blast search against non-redundant GenBank | | BlastN search against *B. glabrata* ESTs | |
| --- | --- | --- | --- | --- | --- | --- | --- | --- | --- |
|  |  |  |  |  |  | e- value  (% identity)  Length matched (position in sequence) | Similarity [accession number] (Blast search) | e- value  (% identity) | N^o^. ESTs matched |
| Cluster 5 (6)  ZB9451  ZB9471  ZB9793  ZB9800  ZB9826  ZBA099 | **DY523248**  **DY523249**  **DY523250**  **DY523251**  **DY523252**  **DY523253** | 512 | Y / Y / 19bp | 105 aa  (1-314) | H / R-S | 8e-44  (81%)  102aa (4-309bp) | Soma Ferritin from *L. stagnalis* [**CAA40096**] (BlastX) | 0.0  (100%) | 2 |
| Cluster 4 (2)  ZBA100  ZBA101 | **DY523254**  **DY523255** | 760 | Y / Y / 24bp | 147 aa  (1-442) | H / R-S | 2e-17  (83%)  171bp (287-456bp) | Fibrinogen related protein 3.2 precusor from *B. glabrata* [**AY028461**] (BlastN) | 0.0  (100%) | 9 |
| Cluster2 (2)  ZB9450  ZBA103 | **DY523256**  **DY523257** | 656 | N | 93 aa  (1-281) | H / R-S | - | No database match | 0.0  (100%) | 34 |
| Cluster3 (2)  ZB9457  ZBA102 | **DY523258**  **DY523259** | 528 | Y / ?*/ 11bp | 137 aa  (1-412) | H / R-S | - | No database match | 0.0  (100%) | 46 |
| ZB9413 | DY523260 | 465 | Y / Y / 14bp | 77 aa  (1-232) | H / R-S | - | No database match | 2e-84  (92%) | 4 |
| Cluster 1 (6)  ZB9318  ZB9323  ZB9361  ZB9366  ZB9827  ZB9828 | **DY523261**  **DY523262**  **DY523263**  **DY523264**  **DY523265**  **DY523266** | 632 | Y / Y / 20bp | 11 aa  (2-34) | H / S-R | - | No database match | - | 0 |
| ZB9365 | DY523267 | 621 | N | 206 aa  1-621 | H / S-R | - | No database match | - | 0 |
| ZBA105 | DY523268 | 628 | N | 188 aa  (106-667) | H / S-R | 1e-62  (30%)  171aa (502-5bp) | Hypothetical protein C31H1.6b from *Caenorhabditis elegans* [**AAN84855**] (BlastX) | - | 0 |
| ZBA3283 | DY523269 | 343 | Y / Y / 22bp | 42 aa  (1-131) | H / E-U | 0.044  (55%)  37aa (9-119bp) | Non-significant match to mitogen activated protein kinase (MAPK) from Leishmania major [**CAJ06341**] (BlastX) | - | 0 |
| ZBA2946 | DY523270 | 732 | N | 246 aa  (1-732) | HO / U-E | 2e-62  (63%)  203aa (140-733bp) | Serine protease HtrA2 from mouse [**Q9JIY5**] (BlastX) | - | 0 |
| ZB9039 | DY523271 | 330 | N | 70 aa  (15-224) | HO / S-R | - | No database match | - | 0 |
