# Supplementary material for: Identification of genes involved in interactions between Biomphalaria glabrata and Schistosoma mansoni by suppression subtractive hybridization
Source: Mol Biochem Parasitol. 2007 Jan;151(1):18–27. doi: 10.1016/j.molbiopara.2006.09.009 (PMC1852639; doi:10.1016/j.molbiopara.2006.09.009)
Supplement: Supplementary file 2 [file mmc2.doc]

| Clone ID/  Name | Material/ Library found in | RT-PCR result | | | | Primer sequences 5’-3’ |
| --- | --- | --- | --- | --- | --- | --- |
|  |  | Haemocytes  R v S | Haemocytes  E v U | Haemopoietic Organ  R v S | Haemopoietic Organ  E v U |  |
| Cluster 5 /  Ferritin | H / R - S | R > S | U > E | No product | No product | F:GCAGTTTGGTGATTACGTCACC  R:CCCATTGAGTTTGCAGACGA |
| Cluster 4 /  Frep intron | H / R - S | R > S | U > E# | R = S | E = U | F:AGCTTTTGGTGCTGCCTGAA  R:CCTTTAGACAGCAACCCGAATG |
| Cluster2 /  Unknown EST* | H / R - S | R = S |  |  |  | F:CGGCAAACTCAACGGTATTGA  R:GAGAGTTGCCTGTGCTGCTTCT |
| Cluster3/  Unknown EST | H / R - S | R > S | U > E# | R slightly > S | U slightly > E | F:AAATATGACTTTCCGATTGGAGTTG  R:CGATAAGGAAAGGCTGTTGGTATC |
| ZB9413/  Unknown EST | H / R - S | R > S | U > E | No product | No product | F:ACTGATCGACCTCCACCTGAAC  R:TCTGCACCATCCTGGATGG |
| Cluster 1/  Unknown EST | H / S - R | S > R | No product | Controls - No product.  Subtracted S > R | No product | F:TGTGTTTGATGCAGTTTCAATGC  R:TGCCCTCAGTCTCCTACGTTTC |
| ZB9365/  Unknown EST | H / S - R | S > R | No product | Controls - No product.  Subtracted S > R | No product | F:TGGTGATAAATGCTCTGGTAGCTC  R:CAGCAATATAATCAAAGGGCAATG |
| ZBA105/  Hypothetical protein | H / S - R | S > R | U > E | No product | No product | F:ACACGTTGCTCATGTCGAAGAC  R:TCGTTACGTGCTGAGCGTTC |
| ZBA3283/  Putative MAPK-like* | H / E - U | - | E = U | - | - | F:CTCATCCAGATCCTGCTCTCGT  R:CGAGAGAACGGATGAGCCAT |
| ZBA2946/  Serine protease HtrA2 | HO / U - E | R > S | U > E | Controls – R = S  Subtracted S > R | U > E | F:TGGCACAGAAAAGACAAATCGA  R:GAATCCCAGGAGACCAGCAA |
| ZB9039 /  Unknown EST* | HO / S-R | - | - | S = R | - | F:GGCTAACCATGCTGCTGAACTC  R:TCAGTTGTGCTTTTCAACCAAGAC |
