# Supplementary material for: Identification of genes involved in interactions between Biomphalaria glabrata and Schistosoma mansoni by suppression subtractive hybridization
Source: Mol Biochem Parasitol. 2007 Jan;151(1):18–27. doi: 10.1016/j.molbiopara.2006.09.009 (PMC1852639; doi:10.1016/j.molbiopara.2006.09.009)
Supplement: Supplementary file 3 [file mmc3.ppt]

## Slide 1
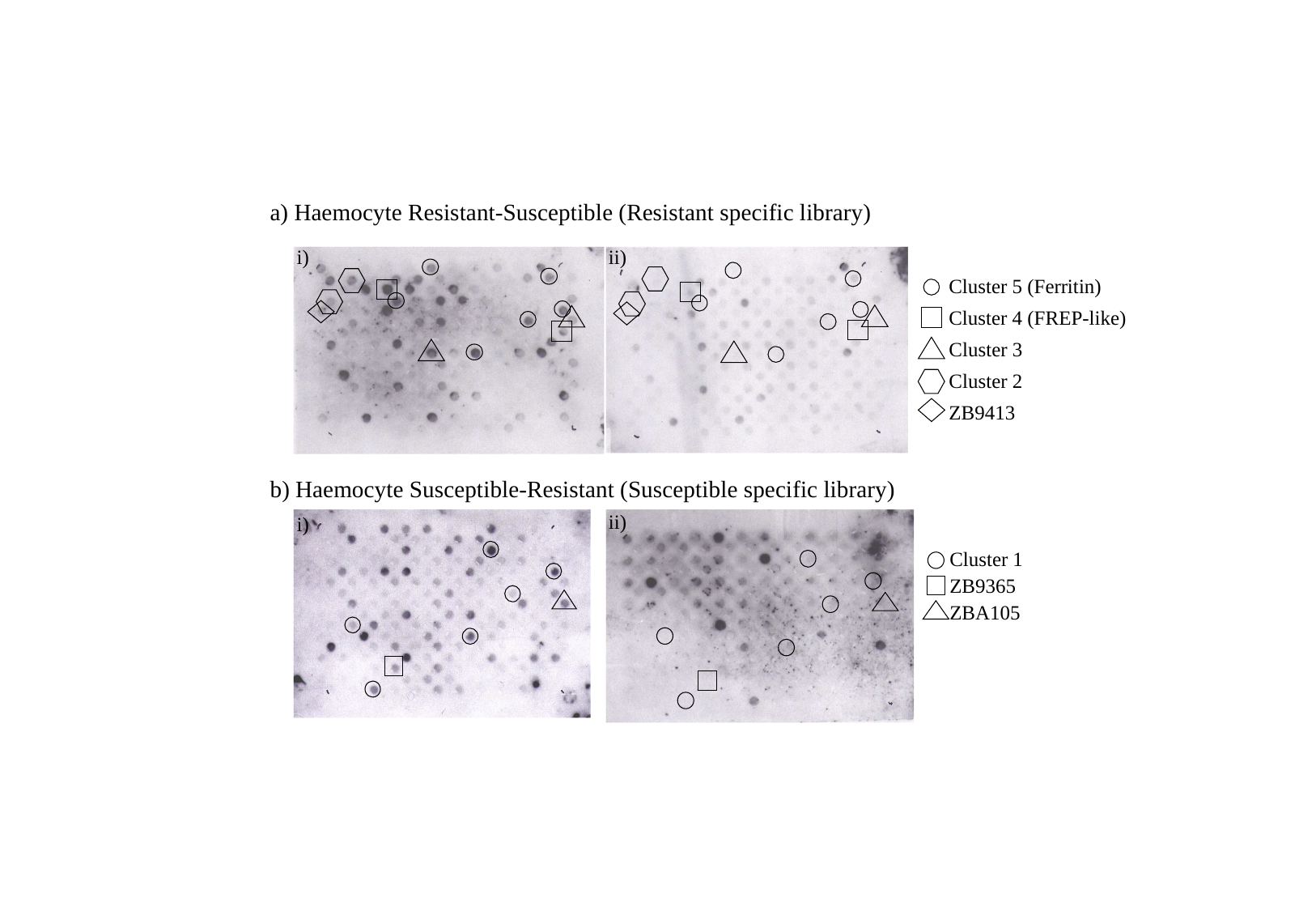

a) Haemocyte Resistant-Susceptible (Resistant specific library)
i)
ii)
Cluster 5 (Ferritin)
Cluster 4 (FREP-like)
Cluster 3
Cluster 2
ZB9413
b) Haemocyte Susceptible-Resistant (Susceptible specific library)
ii)
i)
Cluster 1
ZB9365
ZBA105
